# Imaging of Convection Enhanced Delivery of Toxins in Humans

**DOI:** 10.3390/toxins3030201

**Published:** 2011-03-15

**Authors:** Ankit I. Mehta, Bryan D. Choi, Raghu Raghavan, Martin Brady, Allan H. Friedman, Darell D. Bigner, Ira Pastan, John H. Sampson

**Affiliations:** Division of Neurosurgery, Duke University Medical Center, Box 3807, Durham, NC 27710, USA; Email: bryan.choi@duke.edu (B.D.C.); raghu@therataxis.com (R.R.); mbrady@therataxis.com (M.B.); allan.friedman@duke.edu (A.H.F.); darell.bigner@duke.edu (D.D.B.); pastan@helix.nih.gov (I.P.); john.sampson@duke.edu (J.H.S.)

**Keywords:** convection enhanced delivery, glioblastoma, drug delivery, imaging

## Abstract

Drug delivery of immunotoxins to brain tumors circumventing the blood brain barrier is a significant challenge. Convection-enhanced delivery (CED) circumvents the blood brain barrier through direct intracerebral application using a hydrostatic pressure gradient to percolate therapeutic compounds throughout the interstitial spaces of infiltrated brain and tumors. The efficacy of CED is determined through the distribution of the therapeutic agent to the targeted region. The vast majority of patients fail to receive a significant amount of coverage of the area at risk for tumor recurrence. Understanding this challenge, it is surprising that so little work has been done to monitor the delivery of therapeutic agents using this novel approach. Here we present a review of imaging in convection enhanced delivery monitoring of toxins in humans, and discuss future challenges in the field.

## 1. Introduction

Glioblastoma multiforme (GBM) continues to have a poor prognosis despite aggressive surgical resection and recent advances in radiotherapy and chemotherapy. The median survival after diagnosis of GBM is 14 months [1]. Drug delivery to GBM has been particularly challenging and accounts for some of the difficulty in treating this disease. Since Ehrlich first described the characteristics of blood brain barrier in 1885, it has been known that systemic drugs have limited penetration into the central nervous system. Our current therapeutic options continue to be limited with non-targeted systemic or intrathecal delivery that have side effects of system toxicity, injury to surrounding tissues, and suboptimal drug delivery to the tumor site. The blood brain barrier in particular impedes the delivery of systemically delivered chemotherapeutics by hindering the ability of these agents to cross from the circulation into the tumor cells within the brain [2]. In addition, the highly invasive character of malignant brain tumors confounds our ability to effectively target these diseased tissues using conventional surgical resection and radiation therapy.

**Figure 1:**
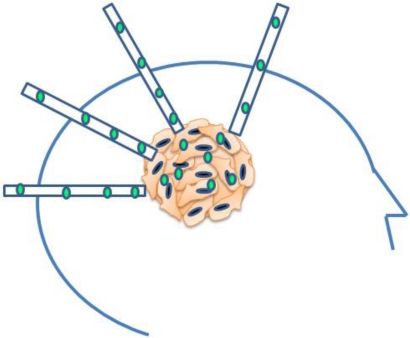
A model of convection enhanced delivery. Catheters are placed into the enhancing tumor to cover the region of effect.

Convection-enhanced delivery (CED) circumvents the blood brain barrier through direct intracerebral application using a hydrostatic pressure gradient to percolate therapeutic compounds throughout the interstitial spaces of infiltrated brain and tumors (Figure 1) [3]. Convection supplements diffusion to greatly enhance distribution of small and large molecular therapeutics [3]. CED also theoretically capitalizes on the presence of an intact blood brain barrier given the advantage of limited egress of therapeutic agent out of the brain, which serves to both enhance drug delivery while reducing the risk of systemic toxicity in humans. CED has a unique property wherein the distribution of therapeutic drug is pressure driven. This phenomena allows for a relatively constant concentration of the immunotoxins for a predictable distance before a drop-off [4], thus limiting neurotoxicity yet providing effective drug therapy to the tumor upon accurate catheter placement [5]. 

The efficacy of CED is determined through the distribution of the therapeutic agent to the targeted region at a sufficient concentration. Catheter placement has been previously noted to have a dramatic effect on drug delivery [5,6] given that leakage of infusate into the interventricular spaces and subarachnoid results in poor drug delivery and distribution [6]. Clinical and preclinical studies have been utilized to determine distribution and delivery of drug into the brain but use differing imaging modalities (MRI, single photon emission computed tomography (SPECT)) and are mostly animal studies that fail to replicate drug distribution in humans [6,7,8,9]. Despite the use of rigorous guidelines and computer simulations, the vast majority of patients fail to receive a significant amount of coverage of the area at risk for tumor recurrence. 

Understanding this challenge, it is surprising that so little work has been done to monitor the delivery of therapeutic agents using this novel approach. Here we present a review of imaging in convection enhanced delivery monitoring of toxins in humans, and discuss future challenges in the field.

## 2. *In Vivo* Imaging of CED Infusate Distribution

The theoretical benefit of pressure-driven infusion is the enhanced distribution of a therapeutic agent at higher concentrations than would typically be achieved by diffusion alone. While mathematical models may predict drug transport as a function of patient-specific parameters, the data needed to validate these models in a noninvasive and real-time fashion can only be obtained with techniques that allow one to measure tissue concentration of a given particle *in vivo*. 

Based largely on indirect measures, many currently available approaches for tracking CED-infused therapies *in vivo* rely on radiographic alterations that occur as a result of either fluid administration or drug-induced effects on tissue. However, as CED continues to gain prominence as a drug delivery platform, the need for more accurate methods of validation will be necessary to objectively assess this approach as an effective means to achieving therapeutic concentrations of a drug in an appropriate target region.

## 3. Albumin-Conjugated Surrogate Tracers

The use of albumin-linked surrogate tracers has been shown in a number of studies to model macromolecular distribution via CED in the primate brain. When linked to either iopanoic acid (IPA) or Gd-diethylenetriamine pentaacetic acid (DTPA), CED-infused albumin tracers can be visualized using conventional noninvasive imaging modalities, CT and MRI respectively. Studies comparing the distribution volume of infused Gd-DPTA-albumin and IP-albumin to that of ^14^C-labeled albumin, as measured by quantitative autoradiography (QAR), have concluded that clinically relevant volumes of macromolecular substrate could be achievable in the brain using the CED approach [10]. 

## 4. MRI Imaging T2 Imaging Changes

More recent investigations have demonstrated that changes in T2-weighted MRIs after treatment in humans can also be used to accurately assess the volume of distribution for therapies administered viaCED. By employing T2-weighted MR in combination with SPECT imaging, Sampson *et al*. showed that three separate reviewers were able to consistently predict intraparenchymal distribution after observing changes in T2-weighted MRI signals 48 h post-infusion [11]. In patients with preexisting MRIs that showed no abnormalities, the emergence of T2 changes following infusion correlated with predicted geometries of drug distribution. Furthermore, in patients who did exhibit preexisting hyperintense signals on T2-weighted MRI, visibly discernable alterations in signal shape and intensity were observed. In this study, ^123^I-HSA was co-infused as a surrogate tracer to compare with T2 changes [12], which revealed inherent limitations with T2-weighted MRI. First, patients demonstrated hyperintense signals on T2-weighted MRI after infusion, though these changes were indistinguishable from those due to pre-existing peritumoral edema secondary to malignant glioma [7]. A second pitfall with T2-weighted MRI was its inability to assess hyperintense signal in gray matter structures [7]. T2-weighted MRI signals could be useful for determining infusate distribution to the inventricular space or subarachnoid space, but its limitations make this modality less than ideal for determining the exact location of infusate when either edema is present or when distribution assessment is attempted throughout gray matter. 

## 5. Gadolinium-Based Liposome Constructs

Gadolinium–diethylene triamine pentaacetic acid (Gd-DTPA) is a widely available MRI contrast agent, however with its small molecular weight (938 Dalton) there are concerns regarding its ability to predict the distribution of larger molecules in CED. Due to this concern, groups have creatively applied this surrogate tracer with some advantage by incorporating it into drug impregnated liposomes [13] or by infusing it into the brainstem where volumes of infusion are low [13,14,15,16]. Liposome constructs, however, require complex manufacturing processes and are also not widely available, and most tumors needing treatment are located in the supratentorial compartment.

## 6. Gadolinium-Bound Albumin

Gadolinium-bound albumin has been utilized due to its larger molecular weight (MW 72,000 D) and has been recently compared in distribution with Gd-DTPA (MW 590 D) at pial and ependymal boundaries [17]. Using a primate model, the authors demonstrated that pial and ependymal boundaries are permeable to both small and large molecular weighted molecules [17]. In addition, they demonstrated that FLAIR MR imaging was more sensitive in detecting Gd-labeled compounds into the CSF during CED [17]. The results of this study seem to demonstrate similar distribution profiles between these agents, but are not consistent with the previous reports of smaller proteins having a different volume of distribution from larger capsids [18].

## 7. Gadolinium Direct Infusion

Because the development of large molecule tracers labeled with gadolinium (Gd) has been problematic, the infusion of low molecular weight Gd-DTPA (Gadolinium conjugated diethylenetriamine penta-acetic acid) is a commonly used MRI contrast agent which can be co-infused with therapies using CED to detect leak and distribution of tracer. 

We have recently demonstrated the ability to define the distribution of larger molecules by imaging dual infusions of Gd-DTPA with iodinated-albumin and the therapeutic drug (MR1-1) [19]. We simultaneously infused patients with supratentorial recurrent malignant gliomas with an EGFRvIII-targeted immunotoxin in combination with ^124^I-HSA (to permit PET imaging) and Gd-DTPA. We demonstrate that Gd-DTPA infusions provide direct information about the distribution of large molecules with high resolution and in combination with fluid-attenuated inversion recovery (FLAIR) imaging, provide additional information about leak into cerebrospinal fluid spaces and resection cavities [19]. 

## 8. Conclusions

CED-infusion approaches offer a promising platform for therapy in patients with GBM. Even though mathematical models are predictive of drug transport and distribution, the ability of *in vivo* imaging to approximate the anatomical location of infusate becomes advantageous in both the real-time monitoring and validation of clinical models. Imaging for CED has evolved from the use of iodine-labeled albumin tracers to direct Gadolinium infusate; however we are still on the frontier of discovery in validating these modalities. Since CED continues to gain prominence as a drug delivery platform in brain tumor patients, the need for more accurate methods of validation grows commensurately. 
